# Role of MicroRNAs in Cancer Development and Treatment

**DOI:** 10.3390/ijms241311058

**Published:** 2023-07-04

**Authors:** Nikolay Mehterov

**Affiliations:** 1 Department of Medical Biology, Medical University-Plovdiv, 4000 Plovdiv, Bulgaria; nikolay.mehterov@mu-plovdiv.bg; 2 Research Institute, Medical University-Plovdiv, 4000 Plovdiv, Bulgaria

MicroRNAs (miRNAs) represent a prominent part of the non-coding landscape of the human genome [1]. Their role is performed on a post-transcriptional level and is accomplished by base pairing mainly with the 3′-UTR of target messenger RNAs (mRNAs). Subsequently, mRNA degradation or translational inhibition occurs, which finally leads to the absence of a protein product. As a part of the human non-coding transcriptome, miRNAs reside in regions which frequently undergo alterations in malignancies. As a consequence, the specific deregulation of miRNAs that control genes involved in the cell cycle, senescence, and apoptosis are observed in all human cancers [2]. Therefore, the specific expression profile of miRNAs in different cancers, together with their stability and easy detection, provides excellent biomarker properties that can be used for diagnosis, staging, and possibly even a wide range of clinical applications.

This Special Issue (SI) presents the potential of miRNAs in the regulation of protein-coding gene expression at a post-transcriptional level in cancer development and treatment. A collection of eleven articles published in this SI offers various examples in the identification, biological role, and the clinical utility of miRNAs in different malignant diseases.

Gliomas are central nervous system tumors with a lethal outcome. Valiulyte et al. examined miR-181a as a potential biomarker for the diagnosis and prognosis of glioma patients. They found that the expression of miR-181a was significantly lower in tumors of grade III and IV. This was associated with IDH1 wild-type gliomas and a shorter overall survival rate. Additionally, they observed a positive correlation between miR-181a levels and the functional status and quality of life of glioma patients. The authors concluded that miR-181a is a unique molecule that plays an important role in gliomagenesis and is associated with changes in a patient’s quality of life [3].

Epithelial ovarian cancer (OvCa) has the highest mortality rate among all gynecological malignancies, mainly due to late diagnosis and a development of resistance to chemotherapy. Stasiak et al. performed miRNA microarray on two doxorubicin (DOX)- and two topotecan (TOP)-resistant variants of the A2780 drug-sensitive ovarian cancer cell line and identified 28 miRNAs that may be related to drug resistance. Among them, miR-125b-5p and miR-935 were upregulated and miR-218-5p was downregulated in the used DOX-resistant cell lines. In both TOP-resistant cell lines, the overexpression of miR-99a-5p, miR-100-5p, miR-125b-5p, and miR-125b-2-3p, along with a decreased expression of miR-551b-3p, miR-551b-5p, and miR-383-5p were observed. An analysis of the targets showed an inverse correlation between the selected miRNAs and important drug-resistant genes, such as the collagen type I alpha 2 chain (COL1A2), protein tyrosine phosphatase receptor type K (PTPRK), receptor tyrosine kinase—EPHA7, roundabout guidance receptor 2 (ROBO2), myristoylated alanine-rich C-kinase substrate (MARCK), and the ATP-binding cassette subfamily G member 2 (ABCG2) [4].

Pirlog et al. investigated the tumor microenvironment (TME) and miRNA tumor profile in a subset of 51 early-stage (T1 and T2) lung cancer samples. They showed that these tumors were immunologically active with 80.4% moderate-to-high inflammatory infiltrates, represented mainly by CD4^+^ cells, which were localized in the stromal compartment. Interestingly, the abundance of CD4^+^ cells was associated with a consistent downregulation of miR-181a-5p in all lung cancer tumor types and a higher expression in the adjacent normal tissue. In addition, they reported that miR-29b-3p, a tumor suppressor miRNA that is usually downregulated in advanced lung cancer, was induced in the early-stage lung cancer cohort, suggesting a late silencing effect. Finally, they presented novel findings concerning the upregulation of miR-29b-3p and miR-181a-5p in p53 IHC-positive tumors [5].

In another work related to OvCa, Loginov et al. used methylation-specific qPCR to identify aberrantly methylated miRNA genes in 102 primary tumors without and with metastases to lymph nodes, peritoneum, or distant organs and 30 peritoneal macroscopic metastases (PMM). They discovered that 13 miRNA genes (MIR107, MIR124-2, MIR124-3, MIR125B-1, MIR127, MIR129-2, MIR130B, MIR132, MIR193A, MIR339, MIR34B/C, MIR9-1, and MIR9-3) were already hypermethylated at the early stages of OvCa, while the hypermethylation of MIR1258, MIR137, MIR203A, and MIR375 was pronounced in metastatic tumors. MIR148A showed high methylation levels specifically in PMM. Furthermore, the expression levels of six miRNAs were significantly decreased in metastatic tumors in comparison with that of nonmetastatic nature. Among them, miR-203a-3p showed the most significant downregulation of miR-203a-3p and also displayed an inverse expression with epithelial–mesenchymal transition (EMT) drivers such as ZEB1 and ZEB2. They also linked the hypermethylation of MIR130B and MIR9-1 with the greatest relative risk of death [6].

Raimondi et al. investigated the role of miRNAs in osteosarcoma (OS), which is the most common primary malignant bone tumor, mainly occurring in young adults. They discovered that miR-CT3 has a low expression in OS cells when compared with human primary osteoblasts and healthy bone. Moreover, they confirmed that VEGF-A is a directed target of miR-CT3 and showed that its enforced expression in SAOS-2 and MG-63 cells reduced not only the expression of VEGF-A mRNA and protein, but also inhibited OS cell migration and invasion. In addition, they reported that miR-CT3 activated the p38 MAP kinase pathway and reduced the expression of EMT players such as vimentin [7].

Drug resistance, caused by changes in the expression of different drug-resistant genes, is the most common reason for high mortality observed in OvCa. Kazmierczak et al. used miRNA microarray to study the changes in miRNA expression levels in two cisplatin (CIS)- and two paclitaxel (PAC)-resistant variants of the A2780 drug-sensitive OC cell line. They found that miR-125b-5p, miR-99a-5p, miR-296-3p, and miR-887-3p were overexpressed and miR-218-5p, miR-221-3p, and miR-222-3p were weakly expressed in both CIS-resistant cell lines. On the other hand, miR-221-3p, miR-222-3p, and miR-4485 were upregulated and miR-551b-3p, miR-551b-5p, and miR-218-5p were downregulated in both PAC-resistant cell lines, thereby suggesting the existence of drug-resistant controlling mechanisms. A subsequent target identification revealed that several important drug-resistant genes such as protein tyrosine phosphatase receptor type K (PTPRK), receptor tyrosine kinase—EPHA7, Semaphorin 3A (SEMA3A), or the ATP-binding cassette subfamily B member 1 gene (ABCB1) can be regulated by these miRNAs [8].

The review paper by Wu et al. concerns the identification of 32 common miRNAs shared by mammary gland development and breast cancer. They reported that these miRNAs are involved in proliferation, metastasis, invasion, and apoptosis in both processes. Some miRNAs were found to have contradictory roles, possibly due to the fact that a single miRNA can target many genes at once. They proposed that the investigation of miRNAs and their role in mammary gland development may provide valuable information with regard to their role in breast cancer with specific emphasis on breast cancer development and treatment [9].

Mario Morales-Martínez and Mario I. Vega reviewed the tumor suppressor role of miR-7 as an important factor in the development and progression of cancer due to its capability to regulate a large number of genes involved in the process of oncogenesis. Their data supported the function of miR-7 as a prognostic biomarker and therapeutic tool in cancer. They summarized the information of the role of miR-7 in various types of cancer, illustrating its regulation, direct targets, and effects, as well as its possible relationship to the clinical outcome of cancer patients [10].

Cervical cancer is a global health burden that has resulted in ~600,000 diagnosed women and 340,000 female deaths in 2020. Choi and Liu performed a literature search regarding miRNAs expressed during the pre-stage of cervical cancer. They summarized the dysregulated miRNAs in clinical samples from cervical pre-cancer patients and related them to the early transformation process, owing to human papillomavirus (HPV) infection in the cervical cells. They concluded that the expressions of miRNAs in cervical pre-cancerous tissue revealed by different studies seldom agreed with each other, possibly due to the discrepancy found among sample types, samples’ HPV status, expression measurements, and methods for analysis. However, miR-34a, miR-9, miR-21, miR-145, and miR-375 were found to be dysregulated across multiple studies, thus highlighting their biomarker properties for cervical pre-cancer detection [11].

Zsuzsanna Gaál discussed the implications of miRNAs in hematological malignancies as well as their clinical utilization. She reported interesting data with regard to novel opportunities for miRNA-based differential diagnosis, chemoresistance prediction, and the prognostic stratification of acute leukemias. Additionally, she discussed the possibilities to use synthetic oligonucleotides and delivery vehicles as therapeutic modulations of miRNA expression levels. Finally, she summarized the drawbacks of these approaches such as inefficient delivery to specific locations, differences in miRNA expression patterns between pediatric and adult hematological malignancies, and the potential side effects of miRNA-based therapies [12].

Finally, a review by Sbirkov et al. summarized miRNAs’ impact on chemotherapy resistance in adult and pediatric acute lymphoblastic leukemia and chronic lymphocytic leukemia. They focused on the miRNA modulation of particular signaling pathways such as PI3K-AKT, transcription factors such as NF-κB, and apoptotic mediators, all of which are bona fide and pivotal elements orchestrating the survival of malignant lymphocytic cells. Finally, they discussed the attractive strategy of using miRNAs mimics, antimiRs and other molecular approaches as promising therapeutic targets, aiming to improve patients’ responses and survival rates [13].

Altogether, this Special Issue of the International Journal of Molecular Sciences presents a collection of articles that cover a wide scientific area, in which miRNAs in cancer development and treatment are involved. As a Guest Editor, I would like to thank all contributors, peer reviewers, Editors, and MDPI’s publishing team for their efforts. I also hope that this SI will be of interest to the scientific community.

## Data Availability

All the required information is provided in the original articles by each author/authors.
